# Conformation Effect on the Mechanical and Microbiological Behavior of Invisible Orthodontic Aligners

**DOI:** 10.3390/ma17061360

**Published:** 2024-03-16

**Authors:** Juan Carlos Rodríguez Fernández, Francisco Pastor, José María Barrera Mora, Elena Demiquels, Eduardo Espinar, Javier Gil

**Affiliations:** 1 Departamento de Ortodoncia, Facultad de Odontología, Universidad de Sevilla, Avicena s/n, 41009 Sevilla, Spain; jucarofe917@gmail.com (J.C.R.F.); franpastordorado@gmail.com (F.P.); jmbmora@us.es (J.M.B.M.); eespinar@us.es (E.E.); 2 Bioengineering Institute of Technology, Facultad de Medicina y Ciencias de la Salud, Universitat Internacional de Catalunya, 08195 Sant Cugat del Vallés, Spain; edemiquels@uic.es

**Keywords:** polyurethane, invisible aligners, mechanical properties, conformation

## Abstract

Invisible orthodontic aligners are having a great impact on tooth movement in an aesthetic and effective way. Different techniques, models, and clinical aspects have been studied for their proper use. However, the aim of this research has been to determine the effect of the shaping process on mechanical properties and their bacterial behavior. For this study, 40 original polyurethane plates and 40 identical models, obtained by hot forming the original plates, were used. The static tensile mechanical properties were studied with a Zwick testing machine using testing speeds of 5 mm/min at a temperature of 37 °C. The original plate and the aligner have been studied with a creep test by subjecting the samples to a constant tension of 30 N, and determining the elongation using a long-distance, high-resolution microscope at different time periods between 1 and 720 h. Studies of water absorption has been realized with artificial saliva for 5 h. Bacterial cultures of *Streptococcus oralis* and *Actinomyces viscosus* strains were grown on the original plates and on new and used models, to determine the proliferation of each bacterium through metabolic activity, colony-forming units, and LIVE/DEAD assays. The mechanical results showed an increase in the strength of the inserts with respect to the models obtained from 3.44 to 3.95 MPa in the elastic limit and a lower deformation capacity. It has been proven that the transition zone in the creep curves lasts longer in the original plate, producing the rapid increase in deformation at a shorter time (400 h) in the aligner. Therefore, the shaping process reduces the time of dental correction exerted by the aligner. The results of the bacterial culture assays show an increase in the number of bacterial colonies when the aligners have been used and when the polyurethane is conformed due to the internal energy of the model, with respect to the original polyurethane. It has been observed that between the original plate and the aligner there are no statistically significant differences in water absorption and therefore the forming process does not affect water absorption. A slight increase in water absorption can be observed, but after five hours of exposure, the increase is very small.

## 1. Introduction

In 1946, Kesling introduced the concept of the transparent orthodontic appliance through a so-called “dental positioner”. In an analogical way, by segregating the teeth in a plaster model, he made small movements and fixed them with wax to later make the vacuum with a thermoplastic plate [1].

The fabrication of various positioners in order to be able to perform larger dental movements was not predictable in daily clinical practice. Shanks, in 1963, developed a machine to make transparent retainers. Nahoum in 1964 patented his “vacuum contouring appliance”. And Ponitz studied all of the problems faced during their manufacturing. Up to that time, all clear appliances were used as a means of retention for completed orthodontic treatment, or for minor tooth movements for slight relapses [2].

The increase in orthodontic treatment of adult patients has also led to an increase in the demand for invisible orthodontic appliances that can replace the commonly used metal brackets. In order to provide an alternative to fixed brackets and wires for this type of patient, invisible orthodontic aligners have been introduced into our treatment options. The aligners allow for better hygiene without affecting the patient’s chewing and allowing for easy removal [3].

The heightened demand for cosmetic orthodontic procedures within the adult demographic has prompted a notable shift from conventional fixed orthodontic appliances to plastic orthodontics. The reluctance of the aesthetic impact of oral appliances has led to the development of transparent therapeutic alternatives, although fixed orthodontic appliances have always been the backbone of orthodontic biomechanics. This transition is, in substantial part, attributed to the strategic marketing initiatives implemented by prominent aligner companies. These companies employ targeted campaigns, particularly through social networks, directly engaging with the patient demographic [4].

The merits of these innovative aligners over traditional orthodontic modalities encompass heightened levels of comfort and enhanced aesthetic appeal. Nonetheless, it is imperative to acknowledge that these advantages are counterbalanced by a concomitant increase in cost for the patient, a technique dependent on the patient’s cooperation, and limitations in addressing the totality of malocclusions [5]. 

Several types of tooth movement have been described in the bibliography as less predictable in contrast to conventional orthodontic methodologies. These encompass space closure, torque, protrusion, and sagittal changes [6].

In 2007, Paul H-Ling listed the limitations of aligner treatment in malocclusions with crowding or gaps of more than 5 mm, anteroposterior skeletal discrepancies of more than 2 mm, centric and centric occlusion relationship discrepancies, highly rotated teeth (more than 20 degrees), severely mesio-distally tilted teeth (more than 45 degrees), teeth with short clinical crowns, and arches with multiple missing teeth [7]

In this regard, it has been demonstrated that the effectiveness of orthodontic movement obtained by using a thermoplastic appliance is inferior to that produced by a fixed appliance [8]

In 1998, Align Technology, Inc. was established, introducing the product Invisalign. Initially utilized for minor overcrowding or diastemas, its application transitioned to a digital format. Advances in material and computer-aided design have significantly expanded the scope of cases addressable through this technique [9].

Presently, digital fabrication techniques rely on CAD-CAM (Computer-Aided Design and Computer-Aided Manufacturing) technology, following a digital workflow protocol. Initially, a digital image is acquired either directly through an intraoral scanner or indirectly through high-quality polyvinylsiloxane (PVS) impressions of the buccal area, which are subsequently digitally scanned to generate a digital model in .stl format. The planned tooth movements are mapped out using digital CAD platforms, generating sequential virtual models depicting the teeth’s desired positions for each stage. Utilizing CAM technology, 3D models of each virtual setup are then produced through either subtractive (milling) or additive (3D printing) manufacturing techniques [10].

The latest published studies show that advances in 3D-printing technology as well as in design programs allow the possibility of directly printing aligners; although, there is currently no commercially available material approved for this purpose [11,12]. Once this is achieved, the mechanical behavior of the printed material in the oral environment should be tested [13].

Aligners’ performance is directly related to the manufacturing process and the composition of the material used to make them. The most widely used is the conventional vacuum thermoforming method that involves molding the thermoplastic material into physical models [11]. Thermoplastic polyurethane (TPU) stands out as an exceptionally versatile polymer known for its superior mechanical and elastomeric properties, as well as its resilience against chemicals and abrasion. Comprising mainly polyols and di- and tri-isocyanates, TPU has been utilized since its early days and continues to be refined for modern applications. Notably, TPU exhibits the unique ability to undergo deformation under load, yet swiftly recover its original shape once the load is removed, thanks to its inherent flexibility [14,15]. 

It is widely known that the composition of oral microflora undergoes significant changes during orthodontic treatment. Conventional orthodontics can lead to an increase in Candida albicans, Streptococcus mutans, and Acidophilic lactobacillus, while also causing a decrease in pH [16].

Similarly, treatment with aligners is linked to bacterial colonization, which may contribute to enamel demineralization. Research has explored the potential of using antibacterial nanoparticles, such as chitosan, in aligners to mitigate bacterial proliferation [17]. Notably, *Streptococcus oralis* is a significant bacterium in the human oral cavity. It is part of the initial colonizers of the tooth surface and is associated with oral health. Coaggregations between *Streptococcus oralis* and other bacteria, such as *Actinomyce*s, play a crucial role in dental plaque formation [18]. *Streptococcus oralis* is a member of the mitis group of oral streptococci and is known to promote biofilm growth by coaggregating in a mutualistic partnership with other early colonizers, contributing to periodontal disease, dental caries, and other oral infections [19]. 

For this reason, this study aims to evaluate the mechanical properties of different aligners and how they can affect the presence of bacterial colonization, specifically *Streptococcus oralis* and *Actinomyces.* Understanding the influence of these bacteria on the mechanical properties of aligners is essential for gaining insights into the potential effects of bacterial colonization on orthodontic devices and oral health.

## 2. Materials and Methods

According to the calculation of experiments, a total of 50 identical clear aligners were studied. These aligners were manufactured by Align Technologies, Inc. (Tempe, AZ, USA) with polyurethane 1,6-hexanedial methylene diphenyl diisocyanate. The thickness of the clear aligners was 0.72 mm. 

### 2.1. Mechanical Assays

**Figure 1:**
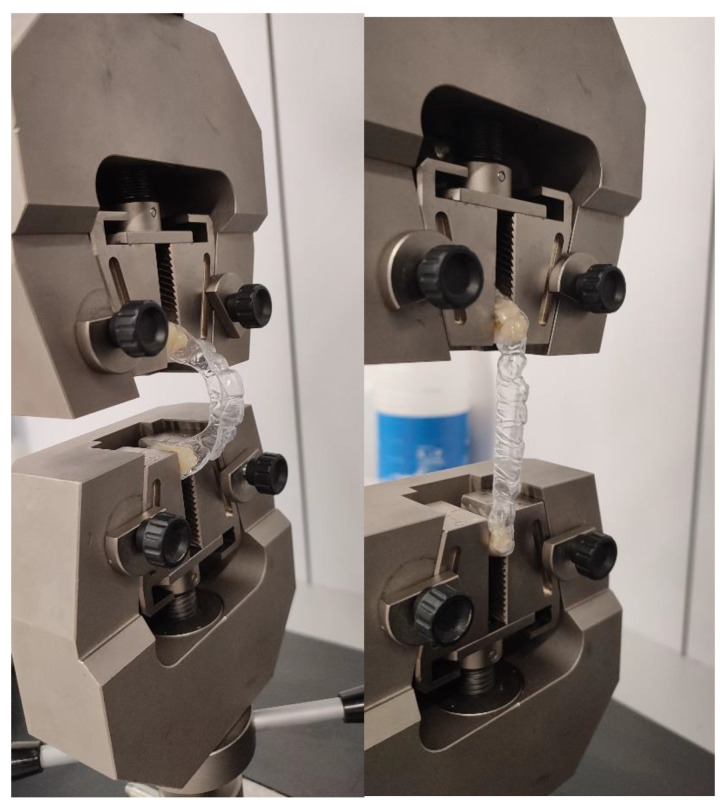
Stress–strain test for determination of mechanical properties.

For the flexural strength assay, 10 specimens for the original plate and 10 specimens for the aligner were used. A universal testing machine (Zwick/Roell, Ulm, Germany) equipped with a 5 kN load cell and a spindle speed of 5 mm/min was used to determine the tensile strength. Prior to testing, the three molars of the aligner were filled with dental resin to create a plane for better gripping of the jaws. The assay system can be seen in Figure 1.

Mechanical tests were carried out at a constant stress of 30 N. The samples were exposed to a constant force and the distance was determined at different time points. With the new distance, the software calculates the elongation. The sensitivity of the distances calculated by the equipment and the software was 0.01 mm and has been used in different research studies that require a high precision [20]. The measurements were performed during one month at different times: 0, 1, 2, 5, 10, 20, 24, 48, 72, 96, 120, 144, 168, 216, 240, 264, 268, 312, 336, 384, 432, 480, 528, 576, 624, 672, 720 h. Elongation was assessed by measuring the change in distance between two specific points on the aligner before and after the experimental period. This evaluation utilized a high-resolution microscope with a long-distance focus; the Q-star system (Oxford Instruments Oxford, UK) [17,18]. 

### 2.2. Scanning Electron Microscopy

The plates (n = 5) and aligners (n = 5) were coated with gold sputtering to make the samples conductive for observation in the scanning electron microscope (JEOL 6400). A 20 KV electron acceleration and a distance of 20 mm between the barrel and the sample were used.

### 2.3. Water Absorption 

The water absorption of the original plate (n = 5) and the aligners (n = 5) was studied at different time points using 35 mm × 35 mm from five different samples. These were stored in a desiccator until a constant weight (Initial weight = W_1_) was reached and then immersed in artificial saliva at 37 °C. The same sample was immersed in artificial saliva, dried superficially, and weighed. The same sample was reintroduced into the artificial saliva and extracted at approximately 24 h, dried, weighed, and immersed again. This process was repeated until the water absorption values were constant, in our case it was 30 days; this test was performed according to ISO 62:2008 [21]. The chemical composition of the artificial saliva is shown in Table 1 [22]. After each time point, they were dried with a cloth and weighed (Second weight = W_2_) on a balance with a sensitivity of 0.00001 g (Sartorius ×1000, Barcelona, Spain). Then, water absorption (WA) was calculated using the following formula [23]

$$\mathrm{WA}=\frac{W2-W1}{W1}\times 100$$

### 2.4. Microbiological Assays

Forty samples for new (n = 20) and used (n = 20) were cut for the different assays, dividing the samples into molars, premolars and incisors. Plaques of the same polyurethane material were also used as samples in this study. Subsequently, all the samples were washed 3 times with PBS.

An inoculum of *Streptococcus oralis* and *Actinomyces viscosus* were prepared, transferring 100 µL of each bacterium into 5 mL of brain heart infusion (BHI), and then incubated overnight at 37 °C for bacterial growth. Subsequently, both inoculums were diluted for the different assays, obtaining an optical density of 0.05. All bacterial cultures were performed under aerobic conditions. In addition, 1 mL of each bacterium were added in each sample into a 48-well plate and incubated at 37 °C for 4 h with agitation to simulate mouth movement.

#### 2.4.1. Colony-Forming Units (CFU)

To quantify the colony-forming units, the supernatants of the different samples from the 48-well plate, mentioned above, were added in 15 mL falcon tubes, washed with 3 mL of PBS, and vortexed. Then, each sample was diluted in different Eppendorf tubes by the serial dilution method between $10^{0}$ and $10^{2}$. Subsequently, 10 µL from each Eppendorf tube were added in duplicate on different agar plates.

#### 2.4.2. Metabolic Activity Assay

To determine the metabolic activity of both bacteria, after 4 h, the supernatant from each well was transferred to another 48-well plate and the samples were washed with 1 mL of PBS. Next, 100 µL of resazurin was added to each well of the plate containing the samples. The 48-well plate was then incubated at 37 °C for 5 min, and then transferred to a black 96-well plate to measure fluorescence in the spectrophotometer (Infinite M nano+, TECAN, Barcelona, Spain) at 560 nm excitation wavelength and 590 nm emission wavelength.

#### 2.4.3. LIVE/DEAD Bacteria Viability

The LIVE/DEAD bacterial viability kit was used only on *Streptococcus oralis* due to its higher intensity, compared to *Actinomyces*. To perform the assay, 3 µL of SYTO9 to stain the live bacteria, 3 µL of propidium iodide to stain the dead bacteria, and 3 mL of PBS were added in a 15 mL falcon tube. All samples were then washed with PBS in different Eppendorf tubes and 100 µL of the previously prepared solution was transferred to all tubes and incubated at 37 °C for 15 min. Subsequently, all of the samples were observed under the confocal microscope (DMi8, Leica, Wetzlar, Germany).

## 3. Results

### 3.1. Mechanical Results 

Table 2 shows the results of the flexural test of the original polyurethane plates and those corresponding to the aligners. The increase in mechanical strength and decrease in deformation caused by forming can be seen.

**Figure 2:**
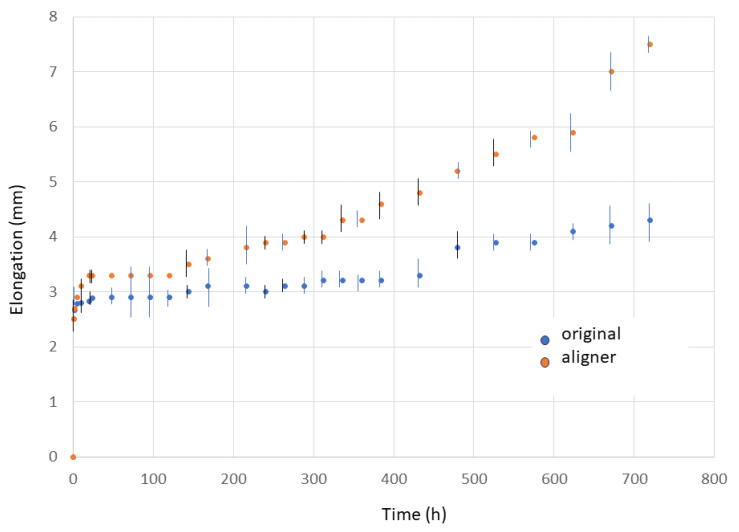
Elongation of original plate and aligner appliance loaded at 30 N in relation to the time in hours.

Figure 2 shows the elongation values with the test time for the original plate and aligner appliance loaded at 30 N.

### 3.2. Scanning Electron Microscope

**Figure 3:**
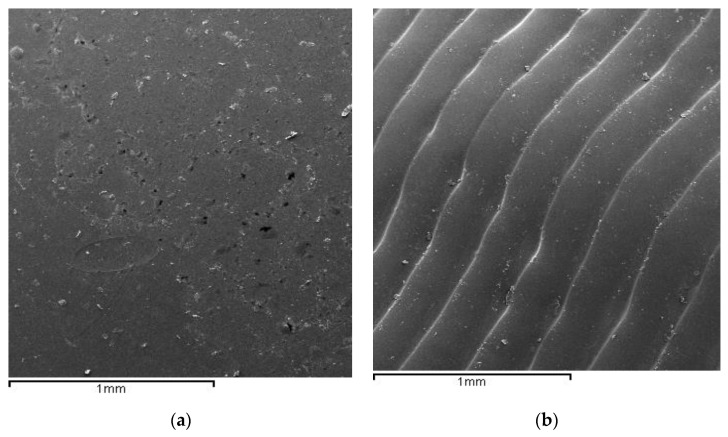
Surface of the polyurethane plate (**a**). Surface of the new aligner (**b**).

Figure 3 shows the structure of the original plate (Figure 3a) and that of the aligners after shaping, where the grooves of the deformations making the shape for dental correction can be seen (Figure 3b).

### 3.3. Water Absorption 

**Figure 4:**
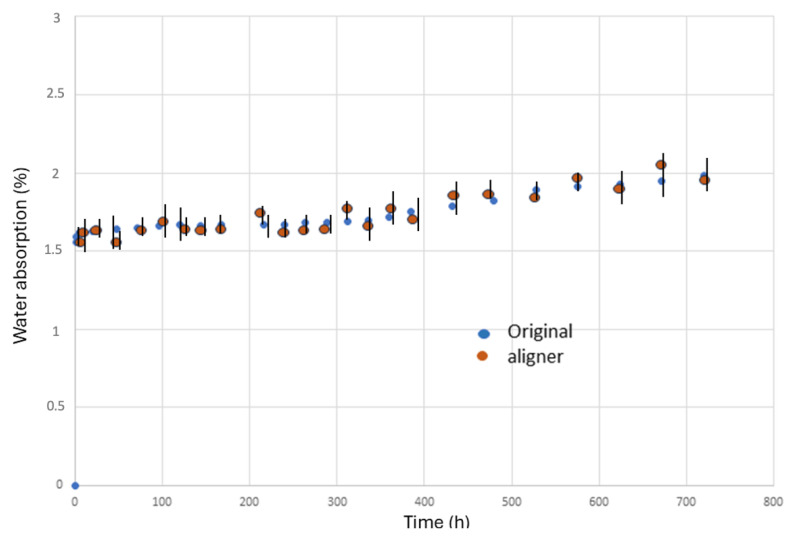
Water absorption up to 1 month.

The results of artificial saliva absorption are shown in Figure 4. As can be seen, the results were constant with the immersion time. No statistical differences in significances have been observed between the original plate and the aligner. Additionally, constant values were maintained from 5 h onwards, as can be seen in Figure 4.

### 3.4. Microbiological Results 

#### 3.4.1. Colony-Forming Units (CFU)

**Figure 5:**
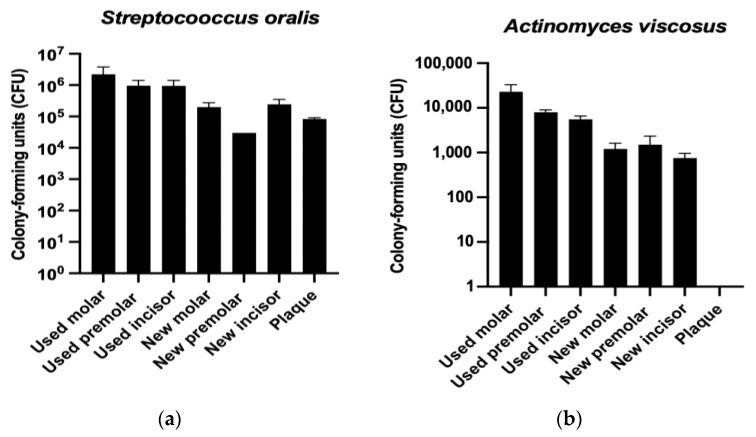
Streptococcus oralis (**a**) and Actinomyces viscosus (**b**) colony-forming units of different used and new dental positions and the original plaque.

Figure 5 shows the formation of *Streptococcus oralis* (Figure 5a) and *Actinomyces viscosus* (Figure 5b) colonies over time, on the different aligner samples. As observed in both strains, the used aligners exhibit a higher number of bacterial colonies compared to the new ones. However, the new aligner samples show higher colony-forming units than the original polyurethane plaques. 

Moreover, it can be also observed that the samples with a higher conformation (molars) exhibit an increase in bacterial colonies compared to those with less conformation. This trend is further supported by the metabolic activity assay represented in Figure 5.

#### 3.4.2. Metabolic Activity Assay

**Figure 6:**
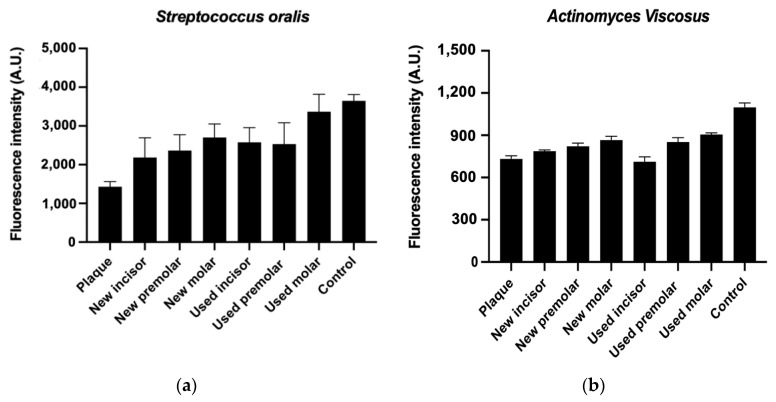
*Streptococcus oralis* (**a**) and *Actinomyces viscosus* (**b**) metabolic activity of different used and new dental positions and the original plaque.

The assessment of the metabolic activity of both *Streptococcus oralis* and *Actinomyces viscosus* strains was conducted over a 4 h period, as illustrated in Figure 6. The results of the assay revealed an increase in the metabolic activity of both bacteria on the used aligners. Notably, the samples that exhibited a more pronounced conformation displayed a higher metabolic activity compared to those with less conformation. These findings suggest that the presence of *Streptococcus oralis* and *Actinomyces viscosus*, along with the observed variations in metabolic activity, may have implications for the mechanical properties of aligners. 

#### 3.4.3. LIVE/DEAD Bacteria Viability

**Figure 7:**
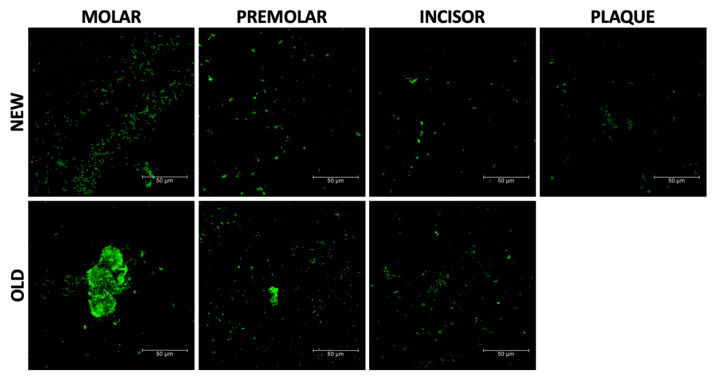
LIVE/DEAD images of new aligners for molar, premolar, and incisor and used aligners (old) for the same teeth and the original plate.

The viability of *Streptococcus oralis* was assessed using the qualitative LIVE/DEAD assay, which involved the observation of live bacteria in green, as illustrated in Figure 7.

The results revealed a higher density of live bacteria on the used aligners compared to the new ones. Additionally, the molar samples exhibited the formation of a biofilm, indicating a greater affinity of bacteria in this area. These findings suggest that the presence of *Streptococcus oralis* and the observed biofilm formation may have implications for the mechanical properties of aligners.

### 3.5. Statistical Analysis

To evaluate any statistically significant differences between the sample groups, data were statistically analyzed using Turkey multiple comparison tests, one-way ANOVA tables, and Student’s *t*-tests. MinitabTM software (Minitab version 13.0, Minitab Inc., Lock haven, PA, USA) was used for all statistical analyses, with asymptotic significance and a 95% significance threshold (*p* < 0.05).

## 4. Discussion

When comparing unused polyurethane plates with formed templates, there is a change in mechanical properties. Polyurethane is a thermoplastic that with temperature causes a semi-crystallization process, which consists of an arrangement of the polymer chains. In addition, the mechanical stresses exerted by the molds on the polyurethane cause the chains to orient themselves in the direction of mechanical stress. This causes the material to lose elasticity by increasing the mechanical stress, making plastic deformation more difficult. Similar results were obtained in Dalaie’s study [22], where the changes in the biomechanical properties of two different types of polyurethanes were compared, and in both materials, there was a decrease in the flexural modulus.

This fact causes a benefit to orthodontic aligners because the material reduces creep and maintains an almost continuous transition zone in the force exerted for tooth movement without the polymer yielding; therefore, the effectiveness of the mechanical load is optimized for tooth movement [24,25]. The choice of the most suitable base material for the construction of orthodontic plastic aligners depends on the knowledge of the chemical and physical properties of each polymeric chemical mixture [26].

The mechanical characteristics of the different polymers are the result of specific manufacturing processes, as opposed to classical orthodontic techniques. The orthodontic force generated by the orthodontic plastic aligner on the dental arch is qualitatively affected by these mechanical properties obtained using an appropriate manufacturing process [26]. For tooth movement to occur, the thermoplastic appliance must be able to continuously transfer controlled orthodontic forces, despite being subjected to continuous and prolonged masticatory loads [27].

When subjected to a mechanical load of 30 N, the materials exhibit an initial deformation of the template immediately upon application of the load at time zero. Subsequently, a marginal increase in elongation is noted, characterized by a relatively flat transition zone. This plateau phase holds particular significance in orthodontic treatment, as it ensures that stress exerted on the tooth does not result in further elongation of the material. Notably, stress on the tooth does not induce additional elongation. However, over longer durations, elongation increases due to the creep phenomenon inherent in polymeric materials [27,28]. This behavior becomes more pronounced when subjected to thermal cycling. After 500 h, a slight alteration in the rate of deformation is observed. When exposed to both the 30 N load and thermal cycling, the plateau diminishes, and elongation begins to escalate significantly after 450 h of treatment. The original plate and aligners exhibit a characteristic creep graph pattern, delineated into three stages [28,29]. When a mechanical load is applied, the first stage corresponds to a sudden increase in deformation.

The second stage, known as the plateau zone, is characterized by a nearly constant mechanical force applied to the teeth. This phase, also referred to as the transition phase, is particularly well suited for the correction of tooth positions. The application of constant, low-intensity forces during this phase is highly favorable for accommodating the surrounding tissue and facilitating the proper apposition and deposition of bone, which are essential for orthodontic movements. The third stage is a stage of gross growth where the aligner may have lost thickness due to constant tension, which has produced a rearrangement of the polymeric chains causing a sudden increase in deformation. The aligner is no longer functional at this stage and cannot move teeth in any way.

From the comparison of the creep curves between the original plate and the aligner, it is possible to observe the reduction in the transition zone of the aligners and an increase in elongation from 400 h onwards, higher than that of the original plate. This fact shows that the forming process impairs the behavior of the original polyurethane plate.

The forming process generates in the polymeric material an increase in the residual internal energy of the template, which causes microbiological activity to be more accentuated, as can be seen in the results of the microbiological cultures carried out on the aligners. Residual energy is a key factor in bacterial proliferation, although changes in topography undoubtedly also play a role [29]. It is well known that areas with concave angles, as well as roughness, facilitate the absorption of bacteria and are preferential areas for the formation of a biofilm. The reliefs obtained in the plates, as could be observed in Figure 2B in the scanning electron microscopy images, could favor bacterial absorption and possible biofilm formation. It is well known that roughness favors bacterial colonization and perhaps these deformations justify the proliferation of the bacteria studied [28].

It has been observed that, between the original plate and the aligner, there are no statistically significant differences in water absorption and therefore the forming process does not affect water absorption. A slight increase in water absorption can be observed, but it is very small after five hours of exposure.

No orientation of the bacteria with the orientation of the polymeric chains is seen in the aligners, but bacteria adsorb in all directions. During the study times, we did not observe an interaction of the bacteria with the polymer; no degradative processes were observed during the 15-day period, which is the time that the aligners normally act during orthodontic movements.

The active bacterial proliferation of the aligners, together with the presence of multiple types of bacteria in the oral cavity, makes it necessary to clean the aligners with bactericidal agents [30]. However, there is a need for further research to develop a transparent coating that is bacteriostatic, as a bactericidal coating may not be ideal due to the presence of bacteria that perform a good mission in the oral cavity. Coatings such as those containing lactoferrin or silver, gold, or copper nanoparticles may not discriminate between different types of bacteria, highlighting the importance of exploring bacteriostatic systems that prevent bacterial adsorption on the surface [31]. Therefore, further work should be conducted on bacteriostatic systems that do not allow adsorption of bacteria on the surface, such as polyethylene glycol coatings, which is an effective bacteriostatic agent [32,33]. The materials from which aligners are made are changing, and new materials are being used. In this work we have studied the most used aligners, but more creep and microbiological studies will be necessary to characterize well the behavior in each of them.

Additionally, it is important to note that while this study has provided valuable insights, there is a limitation in that it focused on a limited number of bacterial strains for biofilm formation. Despite this limitation, this study has revealed that the shaping process increases the mechanical properties of the alginate, but also facilitates bacterial proliferation. 

Even so, scientific evidence has demonstrated that clear aligners are associated with a lower accumulation of plaque and salivary bacteria linked to caries, in comparison to fixed appliances. This difference translates to a reduced frequency and severity of white spot lesions [28]. These findings underscore the potential of clear aligners to offer improved oral health outcomes, making them a favorable option for patients undergoing orthodontic treatment. Further research in this area is essential to continue exploring the impact of clear aligners on oral health and to provide comprehensive insights into their potential benefits.

## 5. Conclusions

The polyurethane-forming process for the construction of invisible orthodontic aligners causes an increase in mechanical resistance and decreases elasticity so that deformation does not increase when tension is applied, when compared to the original polyurethane plates. This fact favors the structural stability of the aligner to affect tooth movement. The internal energy of the process and the striated morphology of the surface of the aligner produced by the forming facilitates the adsorption of bacteria on the surface of the template compared to the original polyurethane. The shaping process causes a reduction in the transition zone of the creep curve and indicates that the activation time of the aligner for tooth movement is approximately 400 h. There are no statistically significant differences in water absorption between the original plate and aligner after 5 h of exposure with artificial saliva. The forming process does not affect water absorption.

## Figures and Tables

**Table 1:** Chemical composition of artificial saliva.

Chemical Composition	g/dm^3^
K_2_HPO_4_	0.20
KCl	1.20
KSCN	0.33
Na_2_HPO_4_	0.26
NaCl	0.70
NaHCO_3_	1.50
Urea	1.50
Lactic acid	Until pH = 6.7

**Table 2:** Mechanical properties of the original plates and aligners. Asterisks indicate statistically significant differences with a *p* < 0.005.

Mechanical Properties	Original Plate	Aligner
Elastic limit (MPa)	3.44 ± 0.15	3.95 ± 0.14 *
Deformation (%)	237 ± 49	207 ± 29 *
Poisson’s ratio	0.492 ± 0.101	0.489 ± 0.162
Elastic modulus (GPa)	0.015 ± 0.002	0.021 ± 0.004

## Data Availability

The data presented in this study are available on request from the corresponding author. The data are not publicly available due to complexity of interpretation.

## References

[1] Kesling H.D. (1946). Coordinating the Predetermined Pattern and Tooth Positioner with Conventional Treatment. Am. J. Orthod. Oral. Surg..

[2] Ponitz R.J. (1971). Invisible Retainers. Am. J. Orthod..

[3] Martorelli M., Gerbino S., Giudice M., Ausiello P. (2013). A Comparison between Customized Clear and Removable Orthodontic Appliances Manufactured Using RP and CNC Techniques. Dent. Mater..

[4] Bichu Y.M., Alwafi A., Liu X., Andrews J., Ludwig B., Bichu A.Y., Zou B. (2023). Advances in Orthodontic Clear Aligner Materials. Bioact. Mater..

[5] Zheng M., Liu R., Ni Z., Yu Z. (2017). Efficiency, Effectiveness and Treatment Stability of Clear Aligners: A Systematic Review and Meta-analysis. Orthod. Craniofac. Res..

[6] Robertson L., Kaur H., Fagundes N.C.F., Romanyk D., Major P., Flores Mir C. (2020). Effectiveness of Clear Aligner Therapy for Orthodontic Treatment: A Systematic Review. Orthod. Craniofac. Res..

[7] Gomez J.P., Peña F.M., Martínez V., Giraldo D.C., Cardona C.I. (2015). Initial Force Systems during Bodily Tooth Movement with Plastic Aligners and Composite Attachments: A Three-Dimensional Finite Element Analysis. Angle Orthod..

[8] Kwon J.-S., Lee Y.-K., Lim B.-S., Lim Y.-K. (2008). Force Delivery Properties of Thermoplastic Orthodontic Materials. Am. J. Orthod. Dentofac. Orthop..

[9] Ke Y., Zhu Y., Zhu M. (2019). A Comparison of Treatment Effectiveness between Clear Aligner and Fixed Appliance Therapies. BMC Oral. Health.

[10] Hartshorne J., Wertheimer M.B. (2022). Emerging Insights and New Developments in Clear Aligner Therapy: A Review of the Literature. AJO-DO Clin. Companion.

[11] Tartaglia G.M., Mapelli A., Maspero C., Santaniello T., Serafin M., Farronato M., Caprioglio A. (2021). Direct 3D Printing of Clear Orthodontic Aligners: Current State and Future Possibilities. Materials.

[12] Panayi N.C. (2023). Directly Printed Aligner: Aligning with the Future. Turk. J. Orthod..

[13] Shirey N., Mendonca G., Groth C., Kim-Berman H. (2023). Comparison of Mechanical Properties of 3-Dimensional Printed and Thermoformed Orthodontic Aligners. Am. J. Orthod. Dentofac. Orthop..

[14] Kasgoz A. (2021). Mechanical, Tensile Creep and Viscoelastic Properties of Thermoplastic Polyurethane/Polycarbonate Blends. Fibers Polym..

[15] Jia Y., Peng K., Gong X., Zhang Z. (2011). Creep and Recovery of Polypropylene/Carbon Nanotube Composites. Int. J. Plast..

[16] Arab S., Nouhzadeh Malekshah S., Abouei Mehrizi E., Ebrahimi Khanghah A., Naseh R., Imani M.M. (2016). Effect of Fixed Orthodontic Treatment on Salivary Flow, PH and Microbial Count. J. Dent..

[17] Taher B.B., Rasheed T.A. (2023). The Impact of Adding Chitosan Nanoparticles on Biofilm Formation, Cytotoxicity, and Certain Physical and Mechanical Aspects of Directly Printed Orthodontic Clear Aligners. Nanomaterials.

[18] Palmer R.J., Gordon S.M., Cisar J.O., Kolenbrander P.E. (2003). Coaggregation-Mediated Interactions of Streptococci and Actinomyces Detected in Initial Human Dental Plaque. J. Bacteriol..

[19] Yadav R.K., Krishnan V. (2022). New Structural Insights into the PI-2 Pilus from Streptococcus Oralis, an Early Dental Plaque Colonizer. FEBS J..

[20] Semiatin S.L., Fagin P.N., Levkulich N.C., Gockel B.T., Antolovich B.F., Crist E.M., Cormier J., Tiley J.S. (2022). The Constant-Stress, Constant-Heating-Rate Test: A Novel Method for Characterizing Transient Mechanical Behavior of Metallic Materials. Metall. Mater. Trans. A.

[21] (2008). Plastics. ISO/TC61/SC6.

[22] Leung V.W., Darvell B.W. (1997). Artificial salivas for in vitro studies of dental materials. J Dent..

[23] Sanjeevi S., Shanmugam V., Kumar S., Ganesan V., Sas G., Johnson D.J., Shanmugam M., Ayyanar A., Naresh K., Neisiany R.E. (2021). Effects of Water Absorption on the Mechanical Properties of Hybrid Natural Fibre/Phenol Formaldehyde Composites. Sci. Rep..

[24] Manero J.M., Gil F.J., Padrós E., Planell J.A. (2003). Applications of Environmental Scanning Electron Microscopy (ESEM) in Biomaterials Field. Microsc. Res. Tech..

[25] Kohda N., Iijima M., Muguruma T., Brantley W.A., Ahluwalia K.S., Mizoguchi I. (2013). Effects of Mechanical Properties of Thermoplastic Materials on the Initial Force of Thermoplastic Appliances. Angle Orthod..

[26] Ryokawa H., Miyazaki Y., Fujishima A., Miyazaki T., Maki K. (2006). The Mechanical Properties of Dental Thermoplastic Materials in a Simulated Intraoral Environment. Orthod. Waves.

[27] Schoenfeld C.M., Conard G.J., Lautenschlager E.P. (1979). Monomer Release from Methacrylate Bone Cements during Simulated In Vivo Polymerization. J. Biomed. Mater. Res..

[28] Rouzi M., Zhang X., Jiang Q., Long H., Lai W., Li X. (2023). Impact of Clear Aligners on Oral Health and Oral Microbiome During Orthodontic Treatment. Int. Dent. J..

[29] Pascual B., Gurruchaga M., Ginebra M.P., Gil F.J., Planell J.A., Goñi I. (1999). Influence of the Modification of P/L Ratio on a New Formulation of Acrylic Bone Cement. Biomaterials.

[30] Tektas S., Thurnheer T., Eliades T., Attin T., Karygianni L. (2020). Initial Bacterial Adhesion and Biofilm Formation on Aligner Materials. Antibiotics.

[31] Rodriguez-Fernandez J.C., Pastor F., Barrera Mora J.M., Brizuela A., Puigdollers A., Espinar E., Gil F.J. (2022). Bacteriostatic Poly Ethylene Glycol Plasma Coatings for Orthodontic Titanium Mini-Implants. Materials.

[32] Raghavan S., Abu Alhaija E.S., Duggal M.S., Narasimhan S., Al-Maweri S.A. (2023). White Spot Lesions, Plaque Accumulation and Salivary Caries-Associated Bacteria in Clear Aligners Compared to Fixed Orthodontic Treatment. A Systematic Review and Meta- Analysis. BMC Oral. Health.

[33] Godoy-Gallardo M., Guillem-Marti J., Sevilla P., Manero J.M., Gil F.J., Rodriguez D. (2016). Anhydride-Functional Silane Immobilized onto Titanium Surfaces Induces Osteoblast Cell Differentiation and Reduces Bacterial Adhesion and Biofilm Formation. Mater. Sci. Eng. C.

